# Supplementary material for: Computer vision analysis to identify episodic flapping in hovering hummingbirds
Source: Biol Open. 2026 May 5;15(4):bio061936. doi: 10.1242/bio.061936 (PMC13225198; doi:10.1242/bio.061936)
Supplement: Supplementary information [file biolopen-15-061936-s1.pdf]

S1 Pausing results

Table S1. True positives (TP), False Positives (FP), and False Negatives (FN) for the classification stage and each analyzed video.

Species	Clade	Sex	TP	FN	FP
Saucerottia saucerottei	Emeralds	U	2	0	1
Amazilia tzacatl	Emeralds	U	0	3	6
Chalybura buffonii	Emeralds	M	1	1	7
Coeligena helianthea	Brilliants	F	5	2	0
Colibri coruscans	Mangoes	F	5	1	0
Coeligena phalerata	Brilliants	F	7	1	2
Ensifea ensifera	Brilliants	M	2	3	1
Eriocnemis cupreoventris	Brilliants	F	3	0	0
Metallura tyrianthina	Coquettes	F	1	2	0
Pterophanes cyanopterus	Brilliants	F	24	3	1
Thalurania colombica (1)	Emeralds	F	2	3	1
Thalurania colombica (2)	Emeralds	F	1	0	0
Thalurania colombica (3)	Emeralds	M	2	0	0
**Total**			55	19	19

Biology Open • Supplementary information

Table S2. Number of pauses, number of wingbeats, pause frequency (number of pauses/number of wingbeats), body mass (Ayerbe-Quiñones 2024) and color under the wing for all the species. The number of wingbeats was taken from Bastidas-Rodríguez et al. (Bastidas-Rodriguez et al. 2024).

Species	# Pauses		# Wingbeat		Pause Frequency		Mean duration		Body mass (g)	Wing color
	Algorithm	Manual	Algorithm	Manual	Algorithm	Manual	Algorithm	Manual		
Saucerottia saucerottei	3	2	75	91	0.04	0.02	0.025	0.022	4.5	Gray
Amazilia tzacatl	6	3	32	25	0.19	0.12	0.016	0.026	5	Gray
Chalybura buffonii	8	2	25	23	0.32	0.09	0.015	0.016	6.8	Gray
Coeligena helianthea	5	7	26	22	0.19	0.32	0.030	0.023	6.7	Black
Colibri coruscans	5	6	18	18	0.28	0.33	0.030	0.025	6.7	Gray
Coeligena phalerata	9	8	59	67	0.15	0.12	0.03	0.023	7.3	Black
Ensifea ensifera	3	3	31	19	0.1	0.16	0.025	0.020	11	Gray
Eriocnemis cupreoventris	3	5	18	142	0.17	0.04	0.056	0.027	5.6	Gray
Metallura tyrianthina	1	3	20	18	0.05	0.17	0.040	0.019	3.8	Gray
Pterophanes cyanopterus	25	27	44	48	0.57	0.56	0.107	0.029	11.2	Blue
Thalurania colombica (1)	3	5	20	28	0.15	0.18	0.023	0.015	4.5	Gray
Thalurania colombica (2)	1	1	10	27	0.1	0.04	0.136	0.016	4.5	Gray
Thalurania colombica (3)	2	2	25	31	0.08	0.06	0.35	0.019	4.5	Gray

Biology Open • Supplementary information
